# Does immune checkpoint inhibitor therapy increase the frequency of adverse reactions to concomitant medications?

**DOI:** 10.1111/cea.14134

**Published:** 2022-03-25

**Authors:** Sean Hammond, Anna Olsson‐Brown, Sophie Grice, Dean J. Naisbitt

**Affiliations:** ^1^ Centre for Drug Safety Science Department of Molecular and Clinical Pharmacology University of Liverpool Liverpool UK; ^2^ ApconiX, Alderley Park Alderley Edge UK

## 1 INTRODUCTION

In recent years, the clinical installation of immune checkpoint inhibitors (ICIs) has proven to be an exceptionally valuable addition to oncological therapy, illustrating superior short‐ and long‐term efficacy in challenging‐to‐treat malignancies and taking on an increasing role across the majority of disease sites. Despite this revolutionary new addition to the oncologist's armamentarium, the “no pain no gain” adage that applies to many of the more established oncological approaches such as chemotherapy still unfortunately appears to hold true in immunotherapy so far. That said, the immune‐mediated mechanism and unpredictability of toxicity has created a new and significant challenge for clinicians and patients alike.

A demonstration of this can be found within the robust association of toxicity and response.^1^ This is presumably attributable to the rather blunt usage of a very precise tool; one can recognise that this approach is currently the systemic deregulation of one or more checkpoints. Thus, in addition to alleviating the negative regulation of tumour‐specific T‐cells by blocking a checkpoint, so too do we deregulate the operations undertaken by that checkpoint by any other pharmacologically accessible cellular components. As a result, the immunological perception of a plethora of antigens is shifted in favour of elicitation. This has given rise to the clinically diverse array of on‐target, off‐tissue reactions that we collectively refer to as immune‐related adverse events (irAEs).^2^ These encompass aberrant adaptive immune responses to endogenous (autoimmune) and exogenous (dietary and commensal organism) derived antigens. Recently, it has come to light that the latter class can be extended to include xenobiotic (namely drug‐related) antigens, which presents as hypersensitivity reactions. One can draw parallels of this heightened immunological state in which the incidence of drug hypersensitivity is increased with the state that must be achieved in individuals who experience multiple drug hypersensitivity; discussed in Ref. [3] The scenario pertaining to checkpoint blockade is probably best described as an immunological drug–drug interaction and is an emerging issue with significant potential clinical impact.

### 1.1 What mechanistic evidence is there for hypersensitivity as subcategory of irAEs?

Evidence to support an enhanced, detrimental immunological response to xenobiotics under the regulatory perturbations imposed by ICIs has been available for much of the time they have been clinically available. Indeed, enhanced *in‐vitro* priming responses to the reactive metabolite of sulfamethoxazole under ICI blockade^4^ and apparent *in‐vivo* drug (amodiaquine) induced idiosyncratic liver injury profiles in PD‐1 knockout, CTLA‐4 blocking antibody treated mice^5^ indicated the nature of this risk. However, the potential relevance of this in the clinical setting has been underappreciated and we have now seen this translate to the clinic, sometimes with fatal consequences.^6^ Rather reassuringly, the mechanism(s) do appear to be in line with what is thought to underlie the efficacy of ICIs in terms of tumour response, evidence for both enhanced priming/recruitment of naive T‐cells,^4^, ^7^ and the enhanced activation of anergised/exhausted memory T‐cells^8^ is now available.

### 1.2 What are the clinical presentations and consequences of these reactions?

Unfortunately, the clinical manifestations of both irAEs and drug hypersensitivity reactions are heterogeneous, have variable latency and lack pathognomonic features.^3^ This represents a challenge, as clinicians in general attribute toxicity to the ICI itself, often unaware of the potential drug hypersensitivity reaction to a concomitant medication.^9^ Given the high frequency of polypharmacy within immune oncology patients, it is feasible that the proportion of irAEs that might in fact be hypersensitivity reactions could be grossly underestimated. This scenario is undesirable on two counts: firstly, the patient might be re‐exposed to the hypersensitivity causing agent, and secondly, the individual may as a result of the reaction, have immunotherapy withheld for extended periods and/or have ameliorative treatment (usually corticosteroids) to the detriment of the intended anti‐malignant efficacy. Furthermore, the burden of risk from corticosteroids and additional immunosuppressive drugs such as hyperglycaemia, opportunistic infection and osteoporosis, is significant and must be navigated as a hallmark of irAE treatment. The standard practice for a clinically significant (CTCAE grade 2 and above) irAE involves holding ICI treatment (sometimes in the form of permanent discontinuation), and usually high‐dose corticosteroid treatment, often followed by a prolonged wean. This is logical in the case of a direct immune toxicity from the dysregulated cells to tissue targets; the endogenous antigens are present throughout and so the deleterious response must be terminated and may recur with readmission of the ICI. However, if the event can be attributed to a hypersensitivity reaction to a concomitant medication, that drug (and with it the implicated antigen) could be removed from the clinical equation with a reduced immunosuppressive burden and possibly permit the reinstatement of a critical systemic anti‐cancer therapy. The possibility that hypersensitivity reactions are responsible for a proportion of what is currently considered irAEs may, therefore, represent an opportunity to both refine the toxicity profile of ICIs, and optimise treatment efficacy.

### 1.3 What are the culprit drugs?

Drug hypersensitivity is generally an exceedingly rare occurrence in the general population. Not all drugs are created equal in this domain and so in the first instance, corroboration of exacerbated toxicity profiles of ICI treated patients in conjunction with drugs with well‐known liabilities for hypersensitivity is an area of interest. Indeed, such cases have begun to be recognised and acted upon; sulfasalazine is an excellent example as it elicits hypersensitivity in the general population at a rate considered to be high but acceptable. However, the incidence of sulfasalazine hypersensitivity appears to be increased to an unacceptable level in ICI treated individuals,^7^ and as a result this drug is effectively informally contraindicated in this patient cohort. Ironically, as the current treatment algorithms for irAEs are lifted from the management of parallel autoimmune diseases, sulfalsalazine formed part of the initial treatment algorithms for ICI‐induced arthralgia. The use of drugs with a pre‐existing, well‐recognised risk for these types of reactions are likely to be problematic in these patients, and the oncology community should be made aware of the potential challenge posed by certain concomitant medications. A review of hypersensitivity literature to identify such compounds might be helpful in terms of identifying therapeutics to closely monitor. Adjunctive therapies such as tyrosine kinase inhibitors also have a proven capacity to elicit such reactions.^6^ It is also important to remember, as demonstrated in our own case report on iodinated contrast media, that previous tolerance of a compound in isolation does not guarantee tolerance once a checkpoint is introduced.^8^ Whilst some combinations will be obviously intolerable, for the vast majority, it might be that the risk‐benefit balance is altered, but ICI treatment is still considered to convey sufficient benefit to warrant treatment. Additionally, pre‐emptive review of concomitant medication could be undertaken as standard to ameliorate the risk. Therefore, wide reassessment of concomitant medications is warranted.

### 1.4 What strategies can we employ to mitigate this toxicity?

**FIGURE 1:**
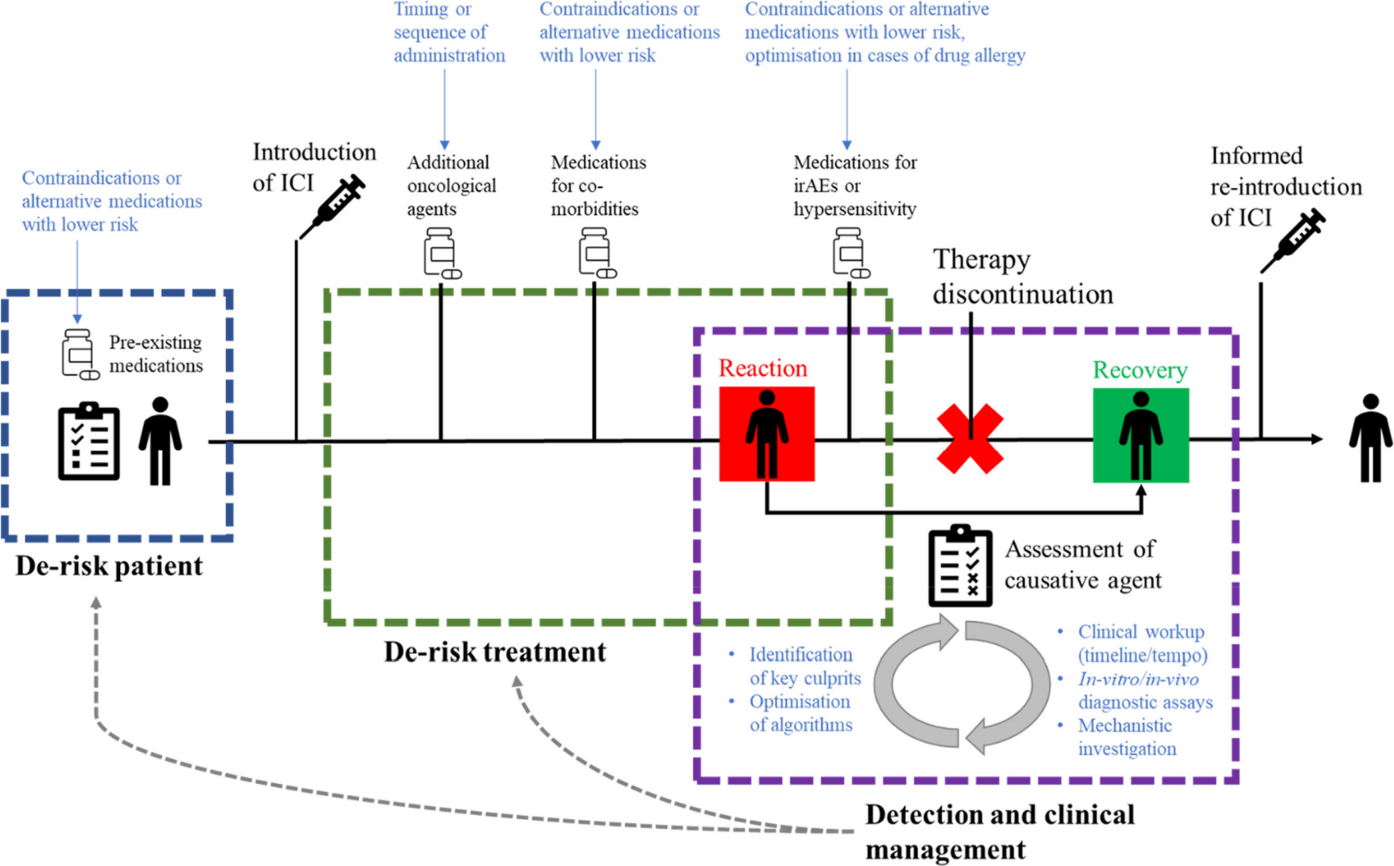
Theoretical patient journey through pre‐treatment screening, administration of ICI therapy, hypersensitivity reaction, recovery and reinstatement of ICI treatment. Potential encounters with concomitant medications are outlined across the three key areas of intervention with suggested areas of investigation outlined in blue

It can be envisaged that there are multiple approaches that could be pursued to address this issue. As outlined in Figure 1, there are several stages at which there is potential to improve. Firstly, de‐risking the patient; it may prove possible to identify which pre‐existing medications might prove problematic prior to commencement of therapy. Next, de‐risking the treatment; where adjunctive therapy is used, immune reactions to the additional agent may compromise the tolerability profile, further, common medications introduced during the course of treatment, for example, antibiotics may well be important. This is particularly relevant when considering irAE therapeutic management strategies and the use of prophylactic antimicrobials following the introduction of ICIs. Finally, there is the matter of accurate and timely diagnoses where these reactions do occur, and along with this, potentially more effective management/treatment algorithms which permit both better resolution of the reaction and optimal cancer treatment.

Broad analysis of which medications ICI patients bring with them may facilitate identification of reactions that occur early on in treatment. A potential example of this was reported in an individual treated with allopurinol and cotrimoxazole who developed toxic epidermal necrolysis following introduction of nivolumab.^9^ For de‐risking of patient and therapy, it is now clear that there will be several drugs for which the toxicity profile in conjunction with ICIs rationalises contra‐indication during an ICI regimen. More subtle refinement (in the form of refinement and referral of concomitant drugs prior to and during ICI treatment) may also be useful in this setting.

For de‐risking the treatment, the adjunctive drug choice has the potential to determine whether therapy is tolerable, for example, dacarbazine combination with ipilimumab is intolerable whilst both agents are tolerable in isolation.^10^ It may be that timing of agents is critical with regards to this, and in time the deconvolution of the optimal sequence/timing of combinatorial strategies will likely prove useful. It is important to remember the pharmacokinetic profile of these biological agents means that the deregulation period will persist for some time after the final treatment. Furthermore, irAEs are observed up to 3 years after ICI treatment has discontinued; thus, there may be a complex interplay with T‐cell memory and naive T‐cell subsets. It is currently unclear as to whether there needs to be active, direct dysregulation to specific T‐cell populations for hypersensitivity, and thus, whether this propensity to hypersensitivity to xenobiotics has a period post ICI following which there is a reduced risk from new con meds or whether the timeline is the same as with self‐antigen (i.e. several years).

There is also the issue of identification of the reactions as hypersensitivity itself, which is critically important in informing the above strategies on which medications are confirmed to be problematic. The emphasis here will be careful clinical evaluation of the concomitant medications the individual has been administered, with critical importance placed on the timeline and tempo of toxicity. Where there is doubt, additional confirmation may be also sought through *in‐vitro*/*in‐vivo* diagnostics. At present, it is not known what the most appropriate diagnostic method may be for this particular form of hypersensitivity reaction, and it is likely that as with more conventional hypersensitivity, the optimal test will be influenced by the reaction type itself. Based on the mechanism of action and reported clinical presentations of reactions reported thus far (apparent predominance of type IV hypersensitivity reactions), one would expect the associated assays (*in‐vivo* patch test, *ex‐vivo* lymphocyte transformation test/cytokine release/other cell‐based assays to be most relevant). As with conventional hypersensitivity, many nuances pertaining to the appropriateness of key assays will apply, for example, the suitability of drug provocation testing will depend on severity of reaction, and thus, associated risk. It is unclear to what degree withdrawal of the xenobiotic would temper the hypersensitivity reaction and to what degree immunosuppression would still be required, but if a temporal relationship could be established then therapeutic withdrawal could enhance clinical diagnostic certainty.

The existence of irAEs have, in themselves, caused significant challenge for the oncological community associated with multi‐organ impact, therapeutic uncertainty and high treatment burdens alongside unpredictable incidence and impact on efficacy. The potential relevance of concomitant medications is largely unconsidered as a potential risk factor or propagator of adverse events. Understanding potential drugs of interest and the differences in management in the presence of a xenobiotic‐induced hypersensitivity has the potential to modify clinical practice in an expanding number of cancer patients, lead to more considered therapeutic combinations within oncology and reduce the incidence of significant toxicity which is blighting the otherwise positive impact of ICI treatment.

## CONFLICT OF INTEREST

The authors declare no conflicts of interest.

## AUTHOR CONTRIBUTIONS

All authors contributed to the conceptualisation and authorship of the manuscript.

## References

[1] Das S , Johnson DB . Immune‐related adverse events and anti‐tumor efficacy of immune checkpoint inhibitors. J Immunother Cancer. 2019;7:306. 31730012 10.1186/s40425-019-0805-8 PMC6858629

[2] Ramos‐Casals M , Brahmer JR , Callahan MK , et al. Immune‐related adverse events of checkpoint inhibitors. Nat Rev Dis Primers. 2020;6:38. 32382051 10.1038/s41572-020-0160-6 PMC9728094

[3] Hammond S , Thomson PJ , Ogese MO , Naisbitt DJ . T‐cell activation by low molecular weight drugs and factors that influence susceptibility to drug hypersensitivity. Chem Res Toxicol. 2020;33:77‐94. 31687800 10.1021/acs.chemrestox.9b00327

[4] Gibson A , Ogese M , Sullivan A , et al. Negative regulation by PD‐L1 during drug‐specific priming of IL‐22‐secreting T cells and the influence of PD‐1 on effector T cell function. J Immunol. 2014;192:2611‐2621. 24510967 10.4049/jimmunol.1302720 PMC3951492

[5] Metushi IG , Hayes MA , Uetrecht J . Treatment of PD‐1(‐/‐) mice with amodiaquine and anti‐CTLA4 leads to liver injury similar to idiosyncratic liver injury in patients. Hepatology. 2015;61:1332‐1342. 25283142 10.1002/hep.27549

[6] Cui W , Cotter C , Sreter KB , et al. Case of fatal immune‐related skin toxicity from sequential use of osimertinib after pembrolizumab: lessons for drug sequencing in never‐smoking non‐small‐cell lung cancer. J Oncol Pract. 2020;16:842‐844. 10.1200/OP.20.00489 32915710

[7] Ford M , Sahbudin I , Filer A , Steven N , Fisher BA . High proportion of drug hypersensitivity reactions to sulfasalazine following its use in anti‐PD‐1‐associated inflammatory arthritis. Rheumatology. 2018;57:2244‐2246. 30107548 10.1093/rheumatology/key234

[8] Hammond S , Olsson‐Brown A , Gardner J , et al. T cell mediated hypersensitivity to previously tolerated iodinated contrast media precipitated by introduction of atezolizumab. J Immunother Cancer. 2021;9(5):e002521. 34049931 10.1136/jitc-2021-002521 PMC8166637

[9] Griffin LL , Cove‐Smith L , Alachkar H , Radford JA , Brooke R , Linton KM . Toxic epidermal necrolysis (TEN) associated with the use of nivolumab (PD‐1 inhibitor) for lymphoma. JAAD Case Rep. 2018;4:229‐231. 29687056 10.1016/j.jdcr.2017.09.028 PMC5909477

[10] Yamazaki N , Uhara H , Fukushima S , et al. Phase II study of the immune‐checkpoint inhibitor ipilimumab plus dacarbazine in Japanese patients with previously untreated, unresectable or metastatic melanoma. Cancer Chemother Pharmacol. 2015;76:969‐975. 26407818 10.1007/s00280-015-2870-0 PMC4612320

